# Etiological Spectrum of Acute Respiratory Infections in Bulgaria During the 2023–2024 Season and Genetic Diversity of Circulating Influenza Viruses

**DOI:** 10.3390/v17020270

**Published:** 2025-02-16

**Authors:** Neli Korsun, Ivelina Trifonova, Diana Pavlova, Yordanka Uzunova, Ivan Ivanov, Daniel Ivanov, Petar Velikov, Silvia Voleva, Tatiana Tcherveniakova, Iva Christova

**Affiliations:** 1 National Laboratory “Influenza and ARD”, Department of Virology, National Center of Infectious and Parasitic Diseases, 1233 Sofia, Bulgaria; trifonova.ivelina@abv.bg (I.T.); diana093@abv.bg (D.P.); iva_christova@yahoo.com (I.C.); 2 Medical Faculty, Department of Internal Diseases, Pharmacology and Clinical Pharmacology, Pediatrics, Epidemiology, Infectious Diseases, and Skin Diseases, University Hospital “Lozenetz”, Sofia University “St. Kliment Ohridski”, 1407 Sofia, Bulgaria; 3 Infectious Diseases Hospital “Prof. Ivan Kirov”, 1431 Sofia, Bulgaria; iri85@abv.bg (I.I.); dannieltiv@gmail.com (D.I.); petar.kr.velikov@gmail.com (P.V.); svoleva@abv.bg (S.V.); taniatcher@gmail.com (T.T.)

**Keywords:** influenza virus, acute respiratory infection, co-infection, whole-genome sequencing, genetic characterization, amino acid substitution, vaccine

## Abstract

Influenza poses a serious threat to both individual and public health. This study aimed to investigate the virological and epidemiological characteristics of influenza infections and to explore the genetic diversity of the circulating influenza viruses. In total, 1886 nasopharyngeal specimens from patients with acute respiratory illnesses were tested against 13 respiratory viruses using a multiplex real-time PCR. Whole-genome sequencing, phylogenetic, and amino acid analyses of representative influenza strains were performed. At least one respiratory virus was detected in 869 (46.1%) patients; 87 (4.6%) were co-infected with two or three viruses. Influenza A(H1N1)pdm09 was the most prevalent virus (16.1%), followed by rhinoviruses (8.1%) and RSV (6.7%). Hemagglutinin (HA) genes of the 74 influenza A(H1N1)pdm09 viruses were categorized in subclades C.1.8, C.1.9, and C.1 within clade 5a.2a and D1, D.2, and D.3 within clade 5a.2a.1. The A(H3N2) viruses analyzed belonged to clade 2a.3a.1, subclades J.2 and J.1. The sequenced B/Victoria lineage viruses fell into clade V1A.3a.2, subclades C.5.6 and C.5.7. Amino acid substitutions in most viral proteins were identified compared with the vaccine strains, including in the HA antigenic sites. This study demonstrated the dominant distribution of the influenza A(H1N1)pdm09 virus among the respiratory viruses studied and the genetic diversity of the circulating influenza viruses.

## 1. Introduction

Viral acute respiratory infections (ARIs) are among humans’ most common infectious diseases. This is largely due to the ease with which the pathogens spread, the short incubation period, the continuously evolving nature of RNA viruses, and the high susceptibility of the population. Among the over 200 known types of respiratory viruses, the most common causes of ARIs are influenza viruses, rhinoviruses (RVs), coronaviruses (CoVs), respiratory syncytial virus (RSV), human metapneumovirus (hMPV), parainfluenza viruses (PIVs), adenoviruses (AdVs), and bocavirus (BoV) [1,2]. Co-infections between respiratory viruses occur and likely impact the disease severity and outcome [3]. Influenza viruses are clinically and epidemiologically significant due to the more severe symptoms they cause and their ability to trigger annual epidemics and periodic (at intervals of 10 to 40 years since 1890) pandemics. Seasonal influenza epidemics affect 3–11% of the entire population, or around a billion people worldwide, with a significant disease burden on young children, older adults, individuals with underlying chronic conditions, immunocompromised people, and pregnant women [4,5]. Every year, about 3–5 million people develop severe illness requiring hospital treatment, and 290,000–650,000 cases of influenza result in death [6].

A notable characteristic of influenza viruses is their high variability and ongoing evolution, driven by a significant mutation rate, rapid replication, and a segmented genome that facilitates gene reassortment among different strains. Influenza viruses evolve with the continuous emergence of new genetic groups, replacing previously circulating strains and increasing genetic and antigenic differences from current vaccine viruses.

Surface glycoproteins of a virus, hemagglutinin (HA), and neuraminidase (NA) are primarily targeted by neutralizing antibodies. During viral replication, minor changes (mutations) occur in the HA and NA genes, leading to substitutions in amino acids. These changes can improve the virus’s ability to evade the immune system, making it more challenging for the host to mount an effective immune response and complicating efforts to control infections. The build-up accumulation of amino acid changes in HA and NA leads to the emergence of new antigenic variants and reduced vaccine effectiveness—a process called antigenic drift. Amino acid substitutions in the immunodominant antigenic sites of the HA1 region of the HA protein, which surrounds the receptor-binding pocket, may enhance the virus’s adaptability for more efficient transmission to humans. These changes primarily contribute to an increased susceptibility to reinfection in individuals who have been previously infected, reduce vaccine effectiveness, and necessitate regular updates to vaccine formulations [7]. For influenza A(H1N1)pdm09, five antigenic sites that produce neutralizing antibodies have been identified in the HA head domain: Sa, Sb, Ca1, Ca2, and Cb. For influenza A(H3N2), five antigenic sites have also been identified: A, B, C, D, and E [8,9]. Type B viruses have three antigenic sites targeted by host immune responses: 120-loop, 150-loop, 160-loop, and 190-helix [10]. An additional viral strategy to evade humoral immunity involves the incorporation of an oligosaccharide at the sequon N X S/T on the HA and NA glycoproteins, with X representing any amino acid other than proline. The glycan chains attached to the HA globular head limit antibody access to the antigenic sites, influence viral antigenicity, and promote immune evasion [11].

In Bulgaria, an ARI surveillance system is utilized to monitor influenza and other respiratory viruses. It consists of a national sentinel network of general practitioners and pediatricians operating in 221 outpatient healthcare facilities located in all 28 regional centers and serving 345,309 people from all age groups (7% of the country’s population (http://grippe.gateway.bg/ (accessed on 11 February 2025)). Physicians from designated sentinel sites collect nasopharyngeal and oropharyngeal swabs from patients with ARI and send them to the National Laboratory “Influenza and ARD” for respiratory virus detection. WHO recognizes the laboratory as the National Influenza Center. It conducts real-time PCR testing on respiratory samples from the sentinel network and severely ill patients hospitalized in various regions of the country.

This study aimed to characterize the virological and epidemiological aspects of influenza in Bulgaria during the 2023–2024 season in relation to other viral respiratory infections. It also sought to analyze the prevalence of co-infections between influenza viruses and other respiratory viruses, as well as to investigate the genetic diversity and molecular evolution of the circulating influenza viruses.

## 2. Methods

### 2.1. Patients and Specimen Collection

Between October 2023 and May 2024, 1886 patients diagnosed with ARIs were recruited from all 28 administrative regions of Bulgaria for the National Influenza Surveillance Program. This cohort included individuals who either received outpatient care or were hospitalized due to their condition. The criteria for study inclusion were aligned with the ECDC definition of an ARI. Specifically, participants were required to present with a sudden onset of symptoms and exhibit at least one of the following four respiratory manifestations: cough, sore throat, dyspnea, or coryza. Additionally, a clinician’s assessment was necessary to determine that the illness was attributable to an infectious process [12]. Nasopharyngeal and oropharyngeal swab specimens were systematically obtained from participants enrolled in the study and promptly transferred to sterile collection tubes containing 2 mL of viral transport medium. Specimens were obtained during the visit to the doctor or within the first 24 h of hospital admission. Following collection, specimens were maintained at a temperature range of 2–8 °C for a maximum of 24 h at the healthcare facilities before being transported in insulated ice packs to the National Laboratory for Influenza and Acute Respiratory Diseases. Specimens were tested within 24 h of collection, and aliquots of the primary samples were stored at −80 °C.

### 2.2. Molecular Detection of Respiratory Viruses

Viral nucleic acids were extracted automatically from 400 μL of each respiratory specimen with an elution volume of 100 μL using a commercial ExiPrep Dx Viral DNA/RNA kit and ExiPrep 16DX instrument (Bioneer, Daejeon, Republic of Korea), according to the manufacturer’s instructions. The extracted viral RNA was tested simultaneously for influenza A/B viruses and SARS-CoV-2 using the FluSC2 Multiplex Real-Time RT-PCR Kit provided by the International Reagent Resource (IRR) (Atlanta, GA, USA) [13]. Real-time RT-PCR was performed to subtype influenza A viruses and the influenza B genetic lineage was determined using the SuperScript III Platinum One-Step qRT-PCR kit (Invitrogen, Thermo Fisher Scientific, Waltham, MA, USA), with specific primers and probes provided by IRR (Atlanta, GA, USA). Amplification was performed using a CFX96 thermal cycler (Bio-Rad Laboratories, Inc., Singapore), according to the protocol recommended by CDC (Atlanta, GA, USA) [14].

The presence of eight common non-influenza respiratory viruses, namely RSV, hMPV, PIV types 1/2/3, RV, AdV, and BoV, was screened using multiplex real-time PCR assays with primers and probes, as previously described [15]. Three PCR mixtures were developed, including the SuperScript III Platinum One-Step qRT-PCR kit and combinations of primers and TaqMan probes labeled with different fluorescent dyes: Mixture 1: AdV + RSV + PIV1; Mixture 2: BoV + RV + PIV2; and Mixture 3: hMPV + PIV3. Positive and negative controls were included in each experiment. The RNAase-P gene was used as an internal positive control during specimen extraction and real-time PCR. For influenza type A and B viruses, positive controls were provided by IRR (Atlanta, USA), while, for other viruses, AmpliRun DNA/RNA Amplification Controls (Vircell, Spain) were used. The following thermocycling parameters were used to detect influenza A/B, SARS-CoV-2, and non-influenza respiratory viruses: reverse transcription: 25 °C for 2 min and then 50 °C for 15 min; initial denaturation: 95 °C for 2 min; amplification for 45 cycles: 95 °C for 15 s and then 55 °C for 30 s. Samples with a cycle threshold (Ct) value < 38 were considered positive. Samples with a cycle threshold (Ct) value < 38 were considered positive.

### 2.3. Sequencing and Phylogenetic Analysis of Influenza Viruses

Influenza-positive samples with high viral load (Ct value ≤ 28) from outpatients and hospitalized patients across various age groups and geographical locations in the country were selected for sequencing. Full-genome sequencing (WGS) of 36 influenza viruses (15 A(H1N1pdm09, 12 AH3N2) and 9 B/Victoria) was performed at the National Laboratory “Influenza and ARD” using a commercial Illumina Microbial Amplicon Prep Kit for Influenza A/B. (Illumina, San Diego, CA, USA). Sequencing was conducted using the Illumina MiSeq system with the 150-cycle v3 reagent kit (Illumina, San Diego, CA, USA). We analyzed the fragment size distribution of DNA libraries using QIAxcel Advanced capillary electrophoresis (Qiagen, Hilden, Germany). Library normalization was performed using the Qubit 4 Fluorometer in conjunction with the Invitrogen™ Quant-iT™ 1X High-Sensitivity (HS) Broad-Range (BR) dsDNA Assay Kit from Thermo Fisher Scientific, Waltham, MA, USA. We utilized the Explify RPIP Data Analysis software (version 2.0.0) available on the BaseSpace platform, provided by Illumina in Cambridge, UK, for genome analysis. In connection with the National Influenza Centers’ responsibility to share recently circulating influenza viruses, we sent influenza virus-positive clinical samples and cell-culture isolates where WGS was performed to the WHO-Collaborating Center in London (UK). Consensus sequences were deposited in the EpiFlu database of the Global Initiative on Sharing All Influenza Data (GISAID). For phylogenetic and molecular analyses, we performed BLAST searches in GenBank and GISAID EpiFlu to retrieve WHO-recommended vaccine strains, reference sequences with known genetic group identities determined by the WHO, and viruses circulating in different European countries during the 2023–2024 season [16].

Multiple sequence alignment was performed using the MUSCLE algorithm implemented in the Molecular Evolutionary Genetics Analysis package, version 11 [17]. The phylogenetic trees for the HA genes were constructed using the maximum likelihood method. The best-fit nucleotide substitution model was the Hasegawa–Kishino–Yano model with a gamma distribution (HKY + G). The tree topology’s reliability was evaluated using 1000 bootstrap replicates. The nucleotide sequences of the Bulgarian influenza viruses analyzed in this study are available in the EpiFlu database of the GISAID, and the accession number list is presented in Supplementary Table S1. In this study, the subclades of influenza viruses were determined by the key amino acid substitutions, as proposed by the report of the ECDC [16].

### 2.4. Deduced Amino Acid Sequence Analysis and Glycosylation Prediction

Translation of the nucleotide code into the amino acid code was performed using BioEdit software (version 7.2). The studied sequences were aligned and compared with the sequence of the vaccine viruses recommended by the WHO for 2023–2024 in the Northern Hemisphere [18] to identify amino acid substitutions in viral proteins. HA-amino acid numbering was performed after the signal peptide was removed.

The NetNGlyc 1.0 web server (https://services.healthtech.dtu.dk/service.php?NetNGlyc-1.0, (accessed on 11 February 2025)) was used to predict putative N-glycosylation sites with a threshold value of 0.5. N-linked glycosylation occurs when the amino acid sequence has N–X–S/T (sequon), where X is any amino acid except proline.

### 2.5. Statistical Analysis

GraphPad Prism version 6.0 was used in statistical analyses. Categorical data were compared using the Chi-square (χ^2^) and Fisher’s exact tests. A *p*-value of less than 0.05 was regarded as statistically significant. Continuous data were presented as either means or medians, depending on the context in which they were analyzed. Statistical comparisons of continuous variables were conducted using the Mann–Whitney U test. Differences with *p* values < 0.05 were considered statistically significant.

## 3. Results

### 3.1. Patient Characteristics

Of the 1886 patients studied, 919 (48.7%) received treatment in primary healthcare facilities, while 967 (51.3%) were hospitalized. The age range of the patients was from 1 month to 90 years, with a median age of 5 years. The study population was divided into five distinct age groups (0–4 years old, 5–14 years old, 15–29 years old, 30–64 years old, and ≥65 years old), comprising 816 (43.3%), 799 (42.4%), 131 (6.9%), 94 (5%), and 36 (1.9%) patients, respectively. Ten (0.5%) patients were of unknown age. Among the study subjects with known sex, 1011 males and 857 females resulted in a male-to-female ratio of 1.18.

### 3.2. Virus Detection

**Figure 1:**
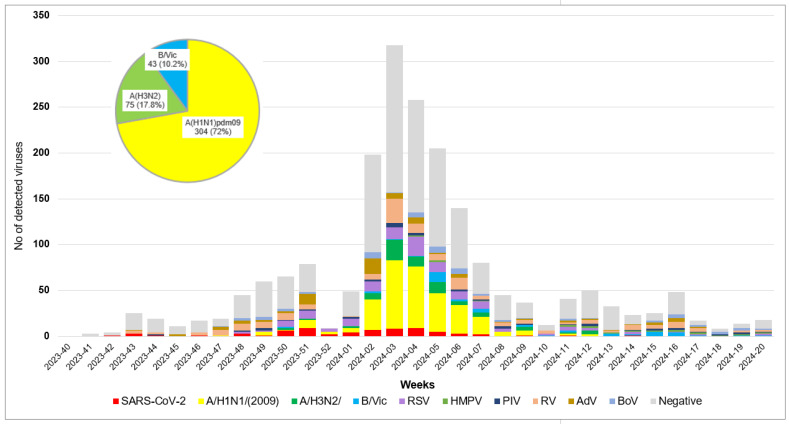
Weekly distribution of detected respiratory viruses during the 2023–2024 season.

The presence of 13 respiratory viruses was prospectively screened in 1886 patients presenting an ARI, and at least one respiratory virus was detected in 869 (46.1%) of them. Among the study patients, 781 (41.4%) were found to be infected with a single virus, 82 (4.3%) were co-infected with two viruses, and 5 (0.3%) were co-infected with three viruses. Influenza viruses were detected in 422 (22.4%) patient samples, of which influenza types A and B accounted for 89.8% (n = 379) and 10.2% (n = 43), respectively. Among the influenza A viruses detected, 304 (72%) were A(H1N1)pdm09 and 75 (28%) were A(H3N2). All the detected influenza type B viruses were assigned to the Victoria lineage. Influenza activity started in week 51/2023 when sentinel detections reached 10% positivity and peaked in week 3/2024. The highest influenza activity was observed in January 2024. In weeks 1–8/2024, the A(H1N1)pdm09 virus dominated, representing ≥60% of the detected influenza viruses. The number of cases positive for influenza type B increased in March 2024. The last positive case (type B) occurred in week 20/2024 (Figure 1).

A total of 69 (3.7%) patients were positive for SARS-CoV-2. Non-influenza respiratory viruses, including RSV (122; 6.7%), hMPV (18; 1%), PIV-1 (1; 0.1%), PIV-2 (13; 0.7%), PIV-3 (27; 1.4%), RV (152; 8.1%), AdV (74; 3.9%), and BoV (61; 3.2%), were detected in 468 (24.8%) patients.

**Figure 2:**
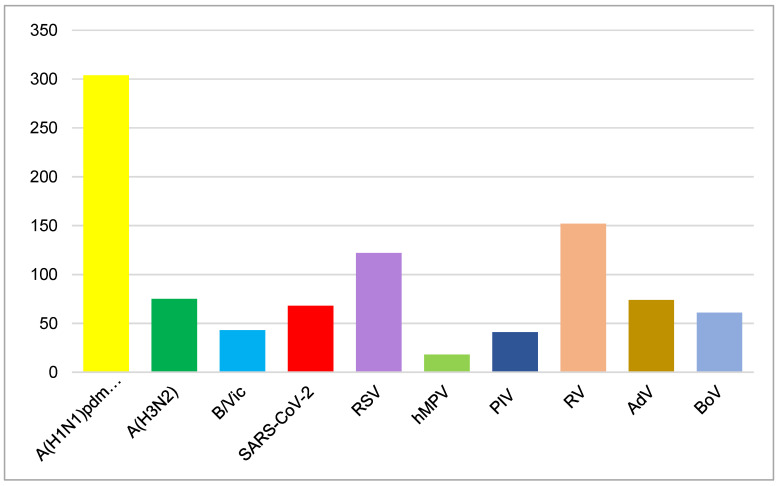
Number of detected respiratory viruses during the 2023–2024 season.

Among the 13 respiratory viruses studied, the influenza A(H1N1)pdm09 virus was most frequently detected, followed by RVs and RSV (*p* < 0.05). HMPV, PIVs, and influenza B/Victoria were identified as having the lowest frequency (<50 cases) (Figure 2).

### 3.3. Epidemiological Characteristics

Viral respiratory infections were observed in all age groups. The proportion of positive cases in the age groups 0–4 years, 5–14 years, 15–29 years, 30–64 years, and ≥65 years was 55.9% (456/816), 39% (312/799), 35.9% (47/131), 40.4% (38/94), and 30.6% (11/36), respectively. Children aged 0–4 years were most frequently affected by respiratory viruses. In this age group, mixed infections between two or three respiratory viruses were most often identified (7.6%, 62/816) (Table 1). The percentages of influenza virus-positive cases among the age groups mentioned above were 23% (188/816), 21.7% (173/799), 17.6% (23/131), 28.7% (27/94), and 25% (9/36). The age group most affected by influenza was 30–64 years (28.7%) followed by +65 years (25%). The average age of the influenza virus-positive patients was 9.9 years (range, 2 months to 87 years; median, 5 years), and 51.4% were male. The predominant A(H1N1)pdm09 virus was more frequently detected among the oldest age group (25%) and 30–64-year-olds (19.1%). The highest rate of influenza B detection (5%) was observed in children aged 5–14 years (*p* < 0.05) and decreased with age. Women (23.6%) were affected slightly more than men (21.5%) by influenza, although the difference was not statistically significant (*p* = 0.2904). In patients with SARS-CoV-2 infections, the positive rate was highest in the older adults (65+ years) (8.3%), and the lowest positive rate was in the 5–14-year-olds (3.1%) (*p* < 0.05). Seasonal non-influenza viruses were most prevalent in the 0–4-year age group, accounting for 51.3%. In the youngest age group, RSV was the most pervasive seasonal non-influenza respiratory virus, followed by RV (*p* < 0.05).

At least one respiratory virus was detected in 388 (42.2%) cases among outpatients, and in 481 (49.7%) among inpatients. Approximately 18.5% (170/919) of outpatients were identified as positive for influenza virus infection, and 26.1% (252/967) of hospitalized patients (*p* < 0.05). The detection rates for influenza A(H1N1)pdm09 virus in outpatients and inpatients were 13.3% (123/919) and 18.7% (181/967) (*p* < 0.05); for influenza A(H3N2) virus—3.6% (33/919) and 4.3% (42/967), and for influenza type B—1.5% (14/919) and 3% (29/967) (*p* < 0.05). SARS-CoV-2-positive cases were detected in 27 (2.9%) outpatients and 41 (4.2%) inpatients.

### 3.4. Clinical Characteristics

**Figure 3:**
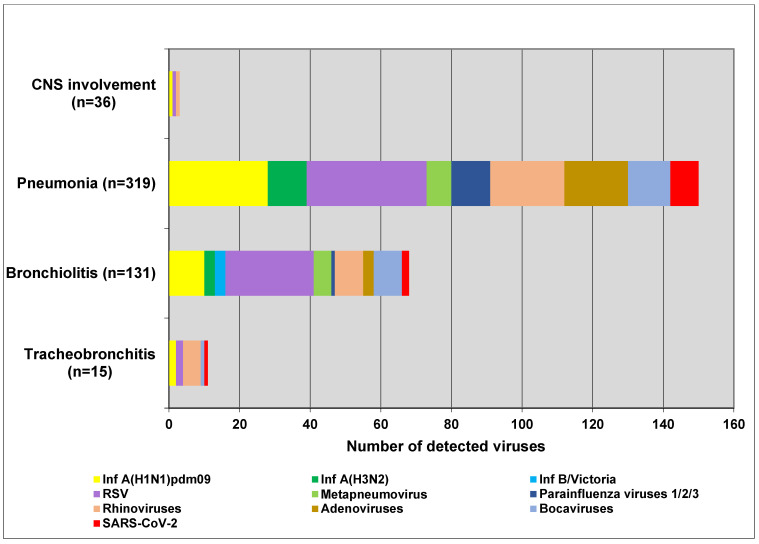
Number of respiratory viruses detected in patients with tracheobronchitis, bronchiolitis, pneumonia, and CNS complications.

Respiratory viruses usually affect the upper respiratory tract and can lead to complications in the lower respiratory tract, heart, and central nervous system. We explored the participation of influenza viruses, SARS-CoV-2, and seasonal non-influenza respiratory viruses in the development of the most common complications—tracheobronchitis, bronchiolitis, pneumonia, and central nervous system involvement (febrile seizures, cerebral edema, viral meningitis, and encephalopathy). The proportions of influenza viruses detected in patients with tracheobronchitis, bronchiolitis, pneumonia, and neurological complications were 13.3% (2/15), 12.2% (16/131), 12.2% (39/319), and 2.8% (1/36), respectively; regarding seasonal non-influenza viruses, the proportions were 53.3% (8/15), 22.9% (30/131), 32.3% (103/319), and 5.6% (2/36), respectively. In SARS-CoV-2-infected patients, the proportions were 26.7%, 1.5%, 2.5%, and 0%, respectively (Figure 3). The detection rates for individual influenza viruses—A(H1N1)pdm09, A(H3N2), and type B viruses—in patients with bronchiolitis were 7.6% (10/131), 2.3% (3/131), and 2.3% (3/131), respectively, and in pneumonia patients were 8.8% (28/319), 3.4% (11/319), and 0% (0/319), respectively. RSV was the most common virus identified in patients with bronchiolitis, accounting for 19.1% (25/131) of cases, and was the second most common cause of pneumonia after influenza viruses in the entire study population, responsible for 10.7% (34/319) (*p* < 0.05). Among the children aged 0–4 years diagnosed with pneumonia, 14.8% (26/176) and 10.8% (19/176) had confirmed RSV and influenza infections, respectively. Among the patients with lower respiratory tract complications due to RSV, 63.3% (38/60) were children under 2 years of age. No deaths were reported in any of the analyzed patients.

### 3.5. Incidence of Co-Infections Between Respiratory Viruses

**Figure 4:**
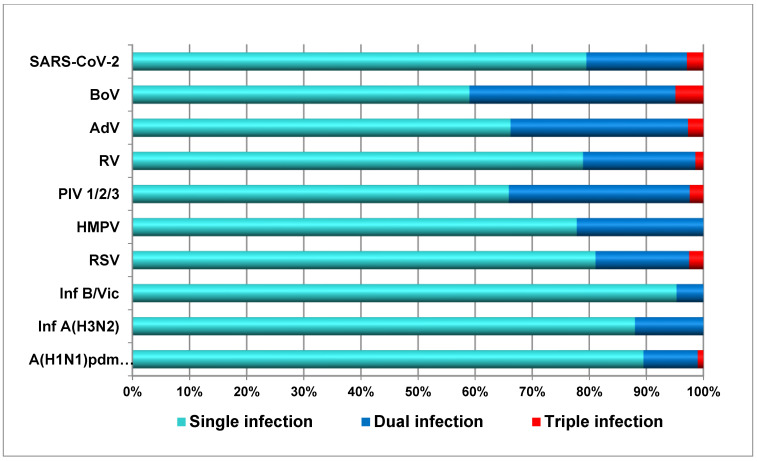
Proportions of single, dual, and triple infections with participation of individual respiratory viruses.

In this study, 87 (4.6%) patients were co-infected with two or three respiratory viruses. Among them, 42 (48.3%) cases of mixed infections involved influenza viruses. There were seven cases of SARS-CoV-2 co-infection with influenza A(H1N1)pdm09 (five cases) and A(H3N2) viruses (two cases). The following seasonal non-influenza viruses were co-detected with influenza: RV (10 cases), BoV (10), AdV (9), RSV (7), PIV3 (2), and PIV2 (1). Figure 4 presents percentages of mono-infections, dual infections, and triple infections for each respiratory virus. Influenza viruses B/Victoria, A(H1N1)pdm09, and A(H3N2) were characterized by the lowest participation in cases of mixed infections—4.7% (2/43), 10.2% (31/304), and 12% (9/75), respectively, while BoV, PIVs, and AdVs were the most frequently involved in co-infections—41% (25/61), 34.1% (14/41), and 33.8% (25/74), respectively. RSV, SARS-CoV-2, RV, and hMPV occupied an intermediate position regarding the share of co-infections with their participation—18.9% (23/122), 20.6% (14/68), 21.1% (32/152), and 22.2% (4/18), respectively.

Table 2 compares the proportions of co-infections for each virus studied in outpatients and hospitalized patients. The proportions of co-infections identified in virus-positive outpatients and inpatients were 4.9% (19/388), and 14.1% (68/481), respectively (*p* < 0.05). The proportions of co-infections involving influenza A(H1N1)pdm09, RSV, PIV type 3, RV, AdV, and BoV were significantly higher in hospitalized patients compared to outpatients (*p* < 0.05). These proportions did not differ considerably for other viruses studied between outpatients and inpatients.

### 3.6. Comparison of Ct Values in Cases of Influenza Virus Coinfections

**Figure 5:**
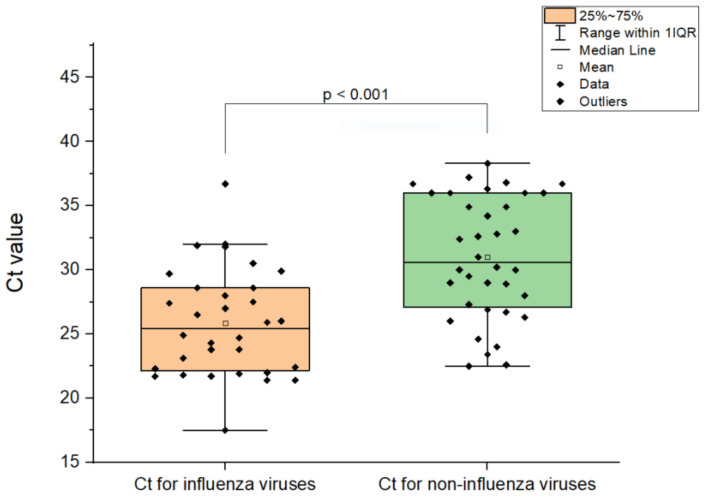
Distribution of Ct values for influenza viruses and other respiratory viruses in cases of co-infections.

The Ct value provides a reliable means to quantify pathogen activity (a low Ct value corresponds to a high viral load in nasopharyngeal swabs). We compared the Ct values for influenza and non-influenza respiratory virus detections in cases of co-infections. In 88% (37/42) of viral co-infections involving the influenza virus, its Ct was lower than that of the concomitantly detected non-influenza viruses (*p* = 0.03). The mean Ct value for influenza virus detections was lower than that of non-influenza virus detections in cases of co-infections (25.9 ± 4.2 and 31.0 ± 4.8, respectively) (Figure 5).

### 3.7. Phylogenetic Analysis of Influenza Viruses

**Figure 6:**
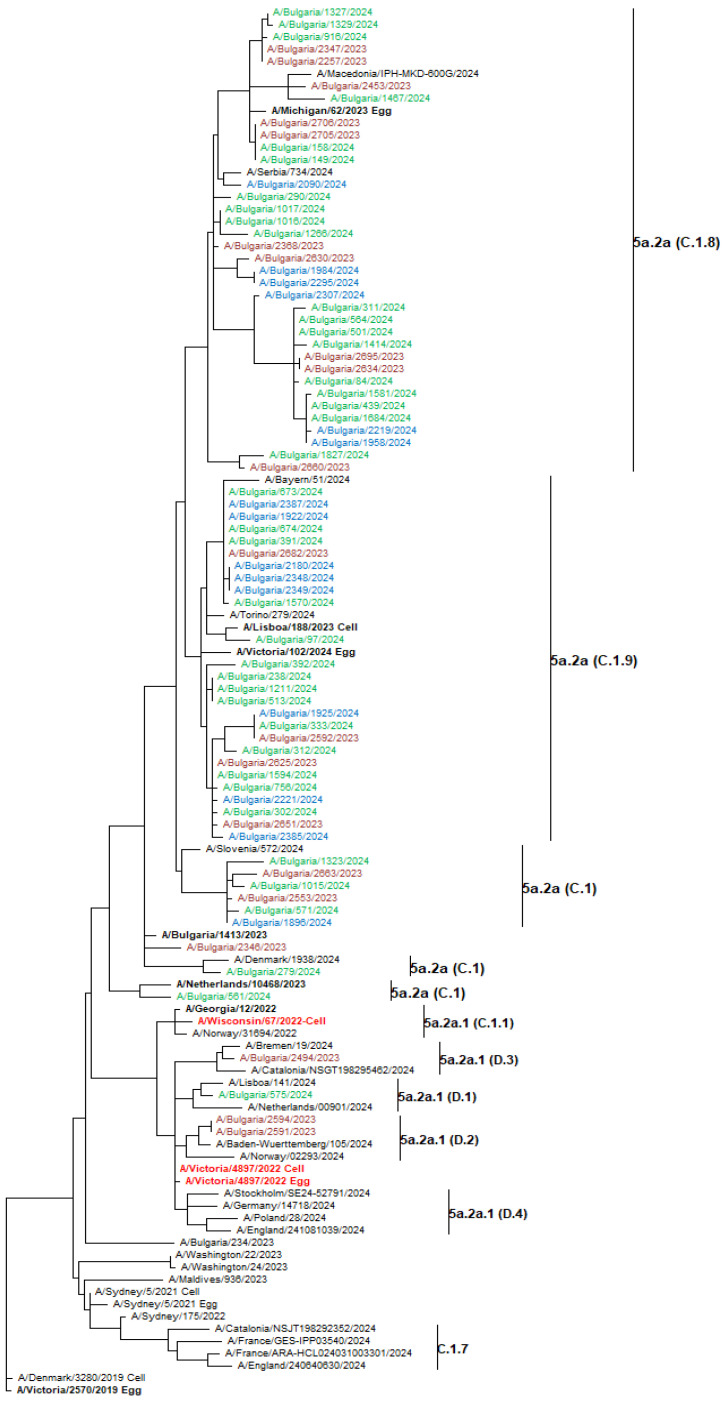
Phylogenetic analysis of the HA nucleotide sequences from influenza A(H1N1)pdm09 viruses circulating in Bulgaria during the 2023–2024 season. The phylogeny tree was generated by the ML method with 1000 bootstrap replicates. WHO-recommended vaccine strains are indicated in red, and reference strains in bold [16]. Bulgarian strains, detected in December 2023, and January and February 2024, are shown in maroon, green, and blue, respectively.

**Figure 7:**
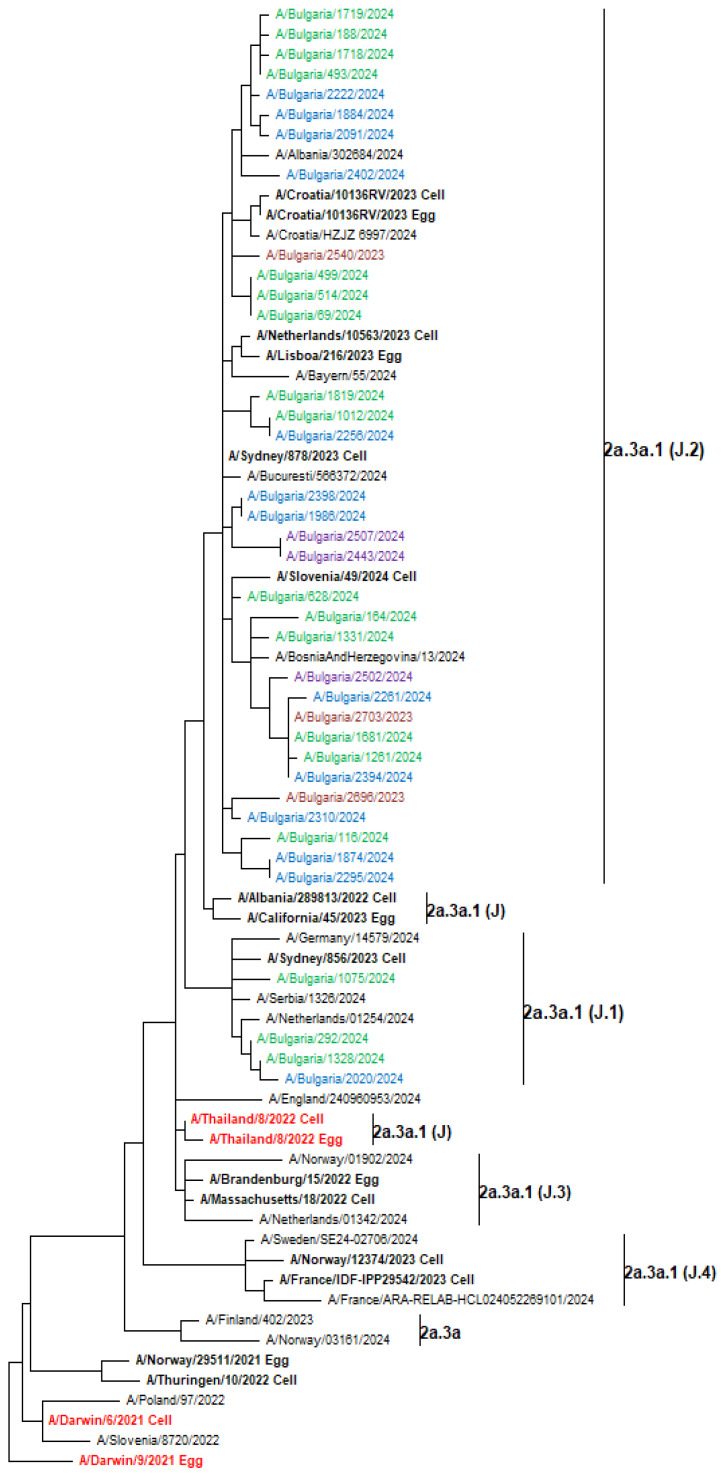
Phylogenetic analysis of the HA nucleotide sequences from influenza A(H3N2) viruses detected in Bulgaria during the 2023–2024 season. The phylogeny tree was generated by the ML method with 1000 bootstrap replicates. WHO-recommended vaccine viruses are indicated in red, and reference strains—in bold [16]. Bulgarian viruses detected in December 2023 and January, February, and March 2024 are shown in maroon, green, blue, and purple, respectively.

**Figure 8:**
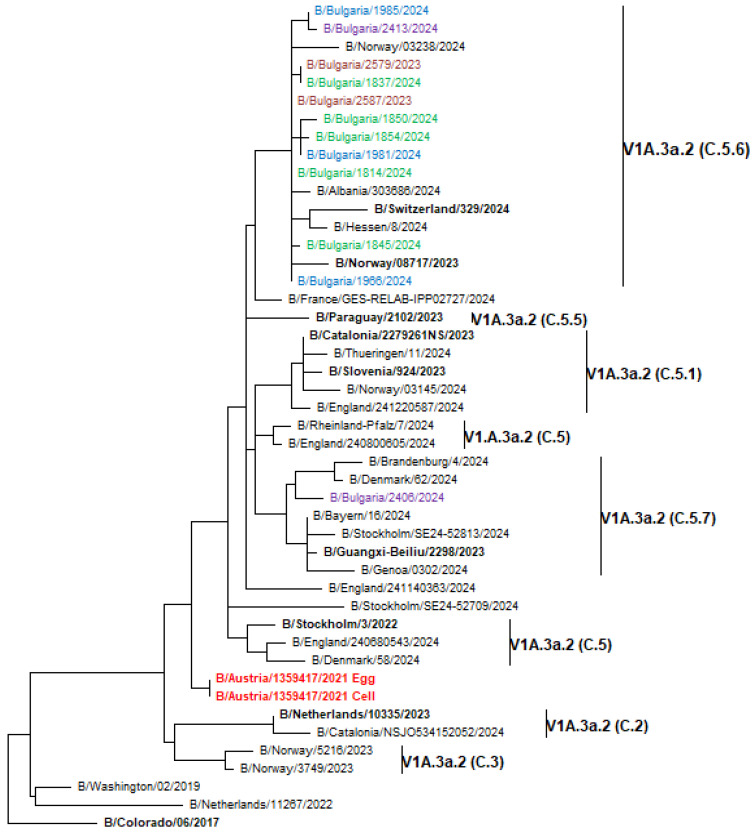
Phylogenetic analysis of the HA nucleotide sequences from influenza B/Victoria lineage viruses circulating in Bulgaria during the 2023–2024 season. The phylogeny tree was generated by the ML method with 1000 bootstrap replicates. WHO-recommended vaccine strains are indicated in red, and reference strains in bold [16]. Bulgarian strains, detected in December 2023 and January, February, and March 2024, are shown in maroon, green, blue, and purple, respectively.

Genetic analysis of circulating influenza viruses was performed due to their widespread distribution and important clinical role. Phylogenetic trees were generated to determine the affiliation of the Bulgarian influenza sequences to the known genetic groups and their genetic relatedness to vaccine strains and viruses circulating in other countries during the same period. The HA genes of the 74 Bulgarian A(H1N1)pdm09 viruses analyzed belonged to two globally distributed genetic clades: 5a.2a (70 strains, 94.6%) and 5a.2a.1 (4 strains, 5.4%). The second clade included the 2023–2024 Northern Hemisphere vaccine strain A/Victoria/4897/2022. Viruses from clade 5a.2a were further categorized into three subclades: C.1.8 (35 strains), C.1.9 (26 strains), and C.1 (9 strains). The representatives of clade 5a.2a.1 fell to subclades D.1 (one strain), D.2 (two strains), and D.3 (one strain), as illustrated in Figure 6 [16]. The 37 A(H3N2) study viruses sequenced were classified in the same clade 2a.3a.1 but fell into two different subclades: J.2, which accounted for the majority of viruses (33 strains, 89.2%) and J.1, which constituted the remaining 4 strains (10.8%) (Figure 7). All 12 B/Victoria-lineage viruses detected in Bulgaria fell into the genetic clade V1A.3a.2, subclade C.5 and clustered together with B/Austria/1359417/2021, which was the vaccine strain for the 2023–2024 NH influenza season. C.5 has further split into two subclades: C.5.6 (11 strains) and C.5.7 (1 strain) (Figure 8).

### 3.8. Amino Acid Polymorphisms in Viral Proteins

The surface glycoproteins HA and NA are under selective immune pressure [19] and evolve rapidly; therefore, they were subjected to detailed molecular analyses. The complete HA and NA amino acid sequences of Bulgarian influenza viruses were compared with those of the WHO NH vaccine strains to identify substitutions that might affect vaccine effectiveness.

#### 3.8.1. A(H1N1)pdm09

When comparing the HA protein of the Bulgarian A (H1N1)pdm09 viruses with the A/Victoria/4897/2022 vaccine strain, 16 amino acid changes were detected in the HA1 polypeptide (globular head) and 6 in HA2 (stem region) (Table 3). The HA1 substitution R223Q was fixed and present in viruses from all genetic groups, and six substitutions were present in subclades 1.8, C.1.9, and C.1 and had a frequency of 94.6%. The substitution T120A was present in subclades C.1.8, C.1.9, and D.3, and S137P in C.1.8 and C.1.9. The other 13 substitutions were specific for individual subclades. Among the specified amino acid substitutions, four affected dominant epitopes: S137P and R142K in Ca, K154R in Sa, and K169Q in Ca1 [20]. No substitutions were identified in the receptor-binding site (RBS): the 190-helix (residues 184–191) [21]. All Bulgarian viruses harbored eight conserved putative *N*-glycosylation motifs in HA (HA1 positions 10, 23, 87, 162, 276, and 287, and HA2 positions 154 and 213). All of these were present in the vaccine strain.

NA sequences of A(H1N1)pdm09 viruses differed from the sequence of the A/Victoria/4897/2022 vaccine strain by 15 substitutions. The substitutions D50N, S200N, and E382G were presented in subclades C.1.8, C.1.9, D.1, and D.2 and showed high frequency: 97.3%, 97.3%, and 98.6%, respectively. Viruses from subclades C.1.8 and C.1.9 carried the common NA substitution I264T with a frequency of 60.8%. The remaining 11 substitutions were specific for the individual subclades (Supplementary Table S3). All Bulgarian viruses harbored eight potential *N*-glycosylation motifs in NA (positions 42, 50, 58, 63, 68, 88, 146, and 235). The substitutions D50N and N73S resulted in a gain of new potential *N*-glycosylation sites. None of the analyzed NA sequences harbored the H275Y amino acid substitution, conferring highly reduced inhibition (HRI) by oseltamivir and peramivir, nor was the Q136K reported in variants with RI by zanamivir [22].

The nucleotide sequences of the remaining genes *PB2, PB1, PA, NP, MP*, and *NS* of influenza A(H1N1)pdm09 viruses were analyzed in comparison to those of the vaccine-derived strain. Several amino acid substitutions were identified in the relevant proteins, as detailed in Supplementary Table S4: two in PB2, two in PB1, five in PA, seven in NP, one in NS1, and three in NEP. M1/M2 had no substitutions. Importantly, no amino acid changes were found in the binding site of the PA inhibitor baloxavir (positions 20, 24, 34, 37, and 38) [22].

#### 3.8.2. A(H3N2)

All 37 analyzed A(H3N2) strains belonged to clade 2a.3a.1 and displayed eight amino acid differences in the HA1 peptide relative to the vaccine virus A/Darwin/9/2021 (Table 3). Representatives of the two subclades J.1 and J.2 carried additional amino acid substitutions: J.2 (10) and J.1 (4). Six substitutions affected antigenic sites A (1), B (2), C (2), and E (1). HA1 substitution I192F was located in the 190-helix. Twelve potential *N*-glycosylation motifs in HA (HA1 positions 8, 22, 38, 45, 63, 96, 122, 126, 133, 165, 246, and 285, and HA2 position 154) were identified. The N96S substitution resulted in a gain, and N122D resulted in a loss of potential *N*-glycosylation site.

Amino acid substitutions were identified in nine positions of the NA protein in comparison with the A/Darwin/9/2021 vaccine strains. Two substitutions, S150H and Y470H, were present in all strains, and R400K was present in both subclades with a frequency of 48.6%. Viruses from the two subclades carried additional amino acid substitutions: J.2 (3) and J.1 (3) (Supplementary Table S3). Nine potential *N*-glycosylation motifs in NA (61, 70, 86, 146, 200, 234, 245, 367, and 463) were identified, with two motifs (146 and 367) located near the enzymatic active site [23].

The following numbers of amino acid substitutions were identified in the remaining viral proteins: PB2 (four), PB1 (two), PA (eight), NP (four), M1 (zero), M2 (four), NS1 (eight), and NEP (one) (Supplementary Table S4). Notably, substitutions in positions 38, 34, and 28 of the PA protein, which are associated with decreased activity of baloxavir, were not observed [22].

#### 3.8.3. B/Victoria

All 12 Bulgarian B/Victoria-lineage viruses belonged to clade V1A.3a.2, subclades C.5.6 and C.5.7 within subclade C.5. The amino acid substitution D197E was identified in HA1 domain in both subclades C.5.6 and C.5.7. The representative of these subclades carried additional amino acid substitutions D129N and E128G, respectively. In type B viruses, the major antigenic regions are the 120-loop (116–137 aa), 150-loop (141–150 aa), and 160-loop (160–172 aa) [24]. The substitutions E128K and D129N were located in the 120-loop and D197E was located in the 190-helix (193–202 aa). No amino acid substitutions occurred in the remaining antigenic epitopes 150-loop and 160-loop [25]. Eleven putative *N*-glycosylation motifs were identified. They were at HA1 positions 25, 59, 145, 166, 233, 304, and 333, and at HA2 positions 145, 171, 184, and 216. *N*-glycosylation motifs 145, 166, and 197 fall into antigenic sites: 150-loop, 160-loop, and 190-helix, respectively.

The B/Victoria-lineage viruses carried a common NA amino acid substitution I459V and additional amino acid substitutions in subclades C.5.6 (three) and C.5.7 (four) (Supplementary Table S3). Four putative *N*-glycosylation motifs were identified at positions 56, 64, 144, and 284. None of the analyzed NA protein sequences displayed substitutions at the active site of NA and their surrounding residues [26].

Substitutions were identified in the remaining viral proteins except for NEP (Supplementary Table S4).

Table 4 summarizes the number of amino acid substitutions in HA antigenic sites compared to the vaccine strains and the number of potential *N*-glycosylation motifs in HA and NA.

## 4. Discussion

In this study, the prevalence, epidemiological, and clinical characteristics of the common respiratory viruses detected in Bulgaria during the 2023–2024 season were presented focusing on influenza viruses. A wide range of respiratory viruses were identified, among which influenza A(H1N1)pdm09 was the most prevalent and SARS-CoV-2 was circulating at a very low level. During the COVID-19 pandemic, extensive non-pharmaceutical measures and travel restrictions targeting SARS-CoV-2 were also effective in reducing the transmission of seasonal respiratory viruses including influenza viruses. During the first two pandemic seasons, the activity of most seasonal respiratory viruses plummeted abruptly worldwide [2]. In 2022–2023, after easing restrictive containment measures, the incidence of seasonal respiratory viruses increased significantly and became comparable to that in the pre-COVID years [27,28,29]. During the study period, most seasonal respiratory viruses studied were detected with varying frequency, and influenza A, RV, and RSV had the highest prevalence. This pattern aligns with findings by Lv et al., who found the highest positive rates for RV, RSV, and influenza A among the tested respiratory viruses [30]. In the USA, during the “tripledemic” in 2023–2024, overlapping peaks of influenza, RSV, and SARS-CoV-2 were a national concern [31]. In our study, the influenza detection rate (22.6%) was close to that in the pre-pandemic season 2018–2019 (34.1%) [32] and significantly greater than that during 2021–2022 (4.2%) [33]. All seasonal influenza viruses were detected, except for the B/Yamagata/16/88 lineage. However, no confirmed B/Yamagata detections have been reported worldwide since March 2020, raising speculation about its possible extinction [34]. In line with other European countries, A(H1N1pdm09) was the most prevalent influenza virus [35]. The seasonality of influenza circulation was similar to that of the pre-pandemic seasons, with a typical peak in January and a second small wave of influenza B at the end of the season [32]. The proportion of positive cases for all studied respiratory viruses was highest in the age group 0–4 years and decreased with age, which is in line with other studies [36]. The detection rate of influenza viruses, in general, and of A(H1N1)pdm09 and B/Victoria, in particular, was higher in hospitalized patients than in outpatients (*p* < 0.05), whereas, for SARS-CoV-2-positive cases, no significant differences were found between outpatients and inpatients (2.9% vs. 4.2%, *p* = 0.1393). A previous study in the WHO European region found a higher proportion of laboratory-confirmed influenza cases in outpatients than in hospitalized patients [37]. Among patients with lower respiratory tract infections, the detection rates for influenza type A (9.9%, 13/131 in cases of bronchiolitis and 12.2%, 39/319 in cases of pneumonia) were higher than those for type B (2.3%, 3/131 in cases of bronchiolitis and 0% in cases of pneumonia) (*p* < 0.05), indicating the higher virulence of type A. Influenza A(H1N1)pdm09 was the most prevalent virus among patients with pneumonia and second among those with bronchiolitis (*p* < 0.05).

The widespread application of multiplex molecular methods in the diagnosis of respiratory tract infections led to the more frequent detection of mixed infections when two or more respiratory viruses are simultaneously present in the same clinical specimen. The prevalence of mixed infections in cases of ARIs varies widely in different studies and depends on several factors such as geographical location, seasonality, patients’ age, social and immune status, and the specific viruses [3]. In our study, 4.6% of patients were co-infected with two or three viruses, and multi-pathogen detections were significantly more frequent in pediatric patients: 7.6% in children 0–4 age and 2.5% in age group 5–14 years. At the same time, no co-infections were found in patients 65+. The rate of viral co-infections in our study was similar to those in Brazilian (7.5%) [38] and Chinese (3.25%) [2] studies and significantly lower than that in the Italian study (37.8%) [36]. In the literature, young age is associated with a higher co-infection rate, which can be explained by an immature immune system leading to increased susceptibility to infections and undeveloped habits for preventing infections [39]. In this study, we showed differences between respiratory viruses in their tendency to co-infect the same individual. Influenza viruses had the lowest co-infection rate, while BoV, PIVs, and AdV were the most frequently involved in co-infections. In line with our results, an Italian study reports the highest co-infection rate for BoV (87.8%), while Flu A has been mainly detected in single infections [36]. In a Spanish study, the proportion of co-detections ranged from 3% for influenza virus to 39% for BoV [40]. Other Spanish researchers found the lowest co-infection rates for FluA, SARS-CoV-2, and hMPV and a high frequency of AdV, RSV, and RV co-infections [41]. Similarly to a study conducted by Siqueira, influenza viruses were most often co-detected with BoV, RV, and AdV [38]. We established that the proportion of co-infections in virus-positive inpatients was higher compared to that in virus-positive outpatients (*p* < 0.05). In addition, the co-infections involving influenza A(H1N1)pdm09, RSV, PIV-3, RV, AdV, and BoV were more common in hospitalized patients than ambulatory patients. Our results were consistent with those of French researchers, who found 4.1 and 93.9 higher frequency of co-infections in hospitalized infants than in primary care for RSV and RV, respectively (*p* < 0.001) [42]. Maio et al. report a higher prevalence of co-infections in children with lower respiratory tract infections compared to those with upper respiratory tract infections [36]. These data suggest that infection with more than one respiratory virus may lead to a more severe clinical course of the disease. However, the clinical significance of mixed infections has not been definitively established and probably depends on the viruses involved. Opinions are divided on whether co-infection with multiple viruses affects disease severity [43,44]. Numerous authors have reported longer hospital stays, a higher risk of hospitalization, increased admission to intensive care units, and even fatalities in patients with confirmed viral co-infections [38,45]. On the other hand, some studies show no link between co-infection and worsened clinical outcomes [46,47]. More research is needed to understand how infections caused by multiple viruses together can impact the seriousness of diseases and the overall results for patients.

The mean value of viral loads, expressed by Ct, for influenza viruses, was higher compared to the mean value of non-influenza viruses in cases of mixed infections, indicating a higher replication rate of influenza viruses and their leading etiological role during co-infection. Virus–virus interactions probably also play a role in these cases where one virus inhibits the replication of another virus through competition for resources, interferon induction, or other immunological mechanisms [48,49].

The genetic characterization of circulating influenza viruses is essential for understanding their evolution and relevance to the effectiveness of vaccines and antiviral drugs. Phylogenetic analysis of Bulgarian 2023–2024 influenza viruses showed significant genetic diversity, especially among the predominant A(H1N1)pdm09 viruses. Influenza A(H1N1)pdm09 accounted for 72% of all influenza viruses detected and belonged to two genetic clades and six subclades: C.1, C.1.8, and C.1.9 within clade 5a.2a, and D1, D.2, and D.3 within clade 5a.2a.1. The majority (94.6%) of Bulgarian A(H1N1)pdm09 viruses belonged to clade 5a.2a whereas the 2023–2024 NH vaccine strain A/Victoria/4897/2022 belonged to clade 5a.2a.1. This discrepancy raises concerns about the 2023–2024 vaccine’s effectiveness in protecting against most circulating viruses. However, according to WHO data, the vaccine strain A/Victoria/4897/2022 matched well with circulating 5a.2a and 5a.2a.1 clades viruses [16]. A similar clade/subclade distribution was found by researchers from Sicily who report modest predicted vaccine effectiveness against the C.1.8 and C.1.9 subclades [35]. In a USA study, 98.9% of the A(H1N1)pdm09 viruses detected in the 2023–2024 season belonged to subclade C.1.1.1 and not a single representative of the most prevalent subclades C.1.8 and C1.9 in our study were identified, demonstrating geography-related variations in the distribution of individual genetic groups of influenza viruses [50]. In total, 16 amino acid changes compared to the vaccine strain were detected in the HA1 polypeptide (globular head) and 6 in HA2 (stem region) including 4 substitutions in HA1 antigenic regions: Sa (1), Ca1 (1), and Ca2 (2) [20]. Both mutations S137P and R142K in Ca2 were the most represented, being found in 82.4% and 94.6% of strains, respectively. No variations were found in the epitopes Sb, Ca1, and Cb or in the receptor-binding site (RBS) located between Sb, Ca2, and Sa. Specific epitopes Sa and Sb are located on the top of the HA protein, while constant sites Ca1, Ca2, and Cb are proximate to the stalk domain [51]. Koel et al. (2015) reported that substitutions in or near the RBS may alter the antigenic properties and adaptability of the virus for enhanced transmissibility [52].

During the study period, the influenza A(H3H2) virus was detected in 17.8% of the influenza viruses circulating in the country and the sequenced strains belonged to two genetic subclades—J.2 (89.2%, 33/37) and J.1 (10.8%, 4/37) within clade 2a.3a.1, represented by influenza A/Thailand/8/2022, the vaccine component for the NH 2024–2025 influenza season. A high frequency (81.2%) of subclade J.2 was also demonstrated in the USA study during the same season [50]. Subclade J.2 was distributed as the globally dominant subclade while subclade J.1 circulated at a significantly lower frequency [16]. From week 40/2023 to week 9/2024, the detection rate of these two subclades in EU/EEA countries was 55% and 34%, respectively [53]. Bulgarian A(H3H2) sequences harbored 22 amino acid changes compared to the vaccine strain, including six substitutions in antigenic sites A (1), B (2), C (2), and E (1), and a substitution located in the 190-helix. Previous studies have shown that antigenic drift variants emerge when at least four substitutions occur simultaneously at two or more antigenic sites [54,55]. Substitutions at antigenic sites A and B, located at the top of HA flanking the RBS, are primarily responsible for antigenic drift [56,57]. No amino acid variations were found at antigenic sites A (position 145) and B (positions 155, 156, 158, 159, 189, and 193) near the RBS, which were responsible for major antigenic changes in A(H3N2) viruses over the period 1968–2003 [58]. Table 4 shows that the A(H3N2) viruses analyzed have higher levels of amino acid polymorphisms in HA antigenic regions and a higher degree of glycosylation compared to A(H1N1)pdm09 and B/Victoria lineage. This explains the higher frequency of generating new antigenic variants in A(H3N2) viruses, the reduced vaccine effectiveness, and the requirement for more frequent updates of the vaccine component [59]. Perofsky et al. suggested a link between increased HA and NA epitope distance between seasons with flu epidemic severity, higher transmissibility, greater A(H3N2) subtype dominance, and increased population susceptibility [60].

During the 2023–2024 season, the majority of globally circulating influenza B/Victoria lineage viruses belonged to clade V1A.3a.2, subclade C.5, which has further split into subclades C.5.1, C.5.4, C.5.6, C.5.7, and basal C.5 [16]. The 12 B/Victoria-lineage viruses, detected in Bulgaria, fell into two subclades: C.5.6 (11 strains) and C.5.7 (1 strain). In contrast to our findings, 75.5% of USA B/Victoria viruses detected were classified as subclade C.5.1 [50]. Two amino acid variations were located at the antigenic site 120-loop and one—at the 190-helix. Previous studies have reported that the 120-loop is one of the sites with the greatest variability and that amino acid substitutions in this region may impact antigenicity and escape from neutralizing antibodies [61,62]. Our findings are in agreement with those of previous studies that showed lower variability and slower evolution of type B viruses [63,64].

The majority of amino acid substitutions identified in the study for the viruses A(H1N1)pdm09, A(H3N2), and B/Victoria lineage presented in Table 3 are new (marked in blue and italic) compared to the strains that circulated in Bulgaria during the previous 2022–2023 season [65].

Overall, a few variations were identified in the *N*-glycosylation motifs compared to those in the vaccine strains. The acquisition or removal of *N*-linked glycosylation can dramatically affect the antigenic properties of viruses [66].

The lack of amino acid changes associated with reduced or highly reduced susceptibility to neuraminidase inhibitors and the PA inhibitor indicates that these antivirals remain a suitable choice for the treatment of influenza [22].

This study had a few limitations. The circulation pattern of influenza viruses was the focus of this study; other respiratory viruses were less well analyzed. The largest number of patients included in the study were children aged 0–4 years, followed by children and adolescents aged 5–14 years. The age groups 30–64 years and especially those over 65 years were poorly represented. Future studies should have a more balanced distribution by age of the enrolled patients. In this study, we observed a higher prevalence of viral co-infections in inpatients compared to outpatients, but we did not examine the clinical data and disease severity in these patient groups. When comparing the Ct values of different viruses, it should be kept in mind that the Ct value is only an indirect method and does not take into account the amplification efficiency of the primers for the different respiratory viruses. Despite these limitations, our study provides a comprehensive overview of the circulation pattern, genomic evolution, diversity, and phylogenetic relationships of influenza viruses during the second post-COVID-19 pandemic season. The data from this study could help public health policymakers in developing effective strategies to control the transmission of influenza viruses and mitigate the severity of future epidemics.

In conclusion, this study showed high activity of the influenza A(H1N1)pdm09 virus and to a lesser extent RVs and RSV in Bulgaria during the 2023–2024 season. Influenza viruses had a relatively low involvement in cases of co-infections. In most cases of mixed infections studied, influenza viruses probably had a leading etiological role due to the higher viral loads. Monitoring cases of influenza virus co-infections with other viral agents is crucial due to their possible implications for disease severity, outcomes, and potential effects on treatment efficacy, vaccination strategies, and infection control measures. Our study revealed notable genetic diversity among the circulating influenza viruses in Bulgaria and offered significant insights into their evolutionary dynamics. Continuous monitoring is essential for enhancing our understanding of influenza evolution and improving public health strategies to effectively manage emerging viruses.

## Figures and Tables

**Table 1:** Age distribution of patients with confirmed viral respiratory mono-infections and co-infections.

Age Group (Years)	Total Positive (%)	Mono-Infections	Dual Infections	Triple Infections
0–4 y (n = 816)	456 (55.9)	394	59	3
5–14 y (n = 799)	312 (39)	293	17	2
15–29 y (n = 131)	47 (35.9)	46	1	-
30–64 y (n = 94)	38 (40.4)	34	4	-
≥65 y (n = 36)	11 (30.6)	11	-	-
without data (10)	4	3	1	-
Total (n = 1886)	869 (46.1)	781 (41.4%)	82 (4.3%)	5 (0.3%)

**Table 2:** Number (%) coinfections involving individual respiratory viruses in outpatients and inpatients.

Viruses	Outpatients		Inpatients		Viruses	Outpatients		Inpatients	
	No Positive	No (%) of Co-Infections	No Positive	No (%) of Co-Infections		No Positive	No (%) of Co-Infections	No Positive	No (%) of Co-Infections
A(H1N1) pdm09	123	7 (5.7%)	181	24 (13.3%) *p* < 0.05	Parainfluenza type 1	0	0 (0%)	1	0 (0%)
A(H3N2)	33	3 (9.1%)	42	6 (14.3%)	Parainfluenza type 2	9	3 (33.3%)	4	1 (25%)
B/Vic	14	1 (7.1%)	29	1 (3.4%)	Parainfluenza type 3	7	0 (0%)	20	10 (50%) *p* < 0.05
SARS-CoV-2	27	5 (18.5%)	41	9 (22%)	Rhinoviruses	78	7 (9%)	74	25 (33.8%) *p* < 0.05
RSV	59	3 (5.1%)	63	20 (31.7%) *p* < 0.05	Adenoviruses	30	6 (20%)	44	19 (43.2%) *p* < 0.05
Metapneumo virus	10	1 (10%)	8	3 (37.5%)	Bocaviruses	23	4 (17.4%)	38	21 (55.3%) *p* < 0.05

**Table 3:** Amino acid substitutions identified in HA protein of influenza viruses A(H1N1)pdm09 (n = 74), A(H3N2) (n = 38), and B/Victoria lineage (n = 12) detected in Bulgaria during the 2023–2024 season (without signal peptide; *+/−* CHO—gain/loss of *N*-glycosylation site; amino acid substitutions marked in blue and italic are new compared to the strains circulating in Bulgaria during the previous 2022–2023 season).

Viruses/Genetic Groups	AA Substitutions	Antigenic Sites	Number of Strains (%)
**A(H1N1)pdm09**			
All strains	R223Q		74 (100)
C.1.9	*N38D*		10 (13.5)
D.1	*R45K*		1 (1.4)
C.1.8	*V47I*		35 (47.3)
C.1.9	*S83F*		11 (14.9)
C.1.8	*I96T*		32 (43.2)
D.2	*R113K*		2 (2.7)
C.1.8, C.1.9, and D.3	*T120A*		62 (83.8)
C.1.8 and C.1.9	S137P	Ca2	61 (82.4)
C.8, C.9, and C.1	R142K	Ca2	70 (94.6)
C.1.8	*K154R*	Sa	13 (17.6)
C.1.9	*K169Q*	Ca1	26 (35.1)
C.1.8	*K208R*		13 (17.6)
C.8, C.9, and C.1	*A216T*		71 (95.9)
C.8, C.9, and C.1	E260D		71 (95.9)
C.8, C.9, and C.1	A277T		71 (95.9)
C.8, C.9, and C.1	D356E		71 (95.9)
D.3	*I372V*		1 (1.4)
C.8, C.9, and C.1	H451N		71 (95.9)
D.2	*V427I*		2 (2.7)
C.1.9	*K480R*		10 (13.5)
C.1.8	*V527I*		6 (8.1)
**A(H3N2)**			
All strains	E50K	C	37 (100)
	D53N	C	37 (100)
	N96S	+CHO	37 (100)
	I140K		37 (100)
	N186D	B	37 (100)
	I192F	B 190-helix	37 (100)
	I223V		37 (100)
	G225D		37 (100)
J.2	*T10M*		5 (13.5)
J.2	*P21S*		6 (16.2)
J.1	*I25V*		4 (10.8)
J.2	*F79L*	E	8 (21.6)
J.2	*R92K*		3 (8.1)
J.2	N122D	A-CHO	33 (89.2)
J.2	*V166L*		5 (13.5)
J.2	*P239S*		8 (21.6)
J.2	*K276E*		33 (89.2)
J.1	*V347M*		3 (8.1)
J.1 and J.2	N378S		21 (56.8)
J.2	*L409I*		8 (21.6)
J.1	*I418V*		4 (10.8)
**B/Victoria lineage**			
All strains	D197E	190-helix	12 (100)
C.5.6	*D129N*	120-loop	1 (8.3)
C.5.7	*E128G*	120-loop	12 (100)

**Table 4:** Number of amino acid substitutions in HA antigenic sites compared to the vaccine strains and number of potential *N*-glycosylation motifs in HA and NA of influenza viruses circulating in Bulgaria during the 2023–2024 season.

Influenza Viruses	Vaccine Strains	Antigenic Sites						*N*-Glycosylation Motifs	
								HA	NA
A(H1N1)pdm09	A/Victoria/4897/2022	Sa	Sb	Ca1	Ca2	Cb	190-helix	8	8
		1	-	1	2	**-**	-		
A(H3N2)	A/Darwin/9/2021	A	B	C	D	E	190-helix	12	9
		1	2	2	-	1	1		
B/Victoria lineage	B/Austria/ 1358417/2021	120-loop	150-loop	160-loop			190-helix	11	4
		2	**-**	**-**			1		

## Data Availability

Data are contained within the article (and its Supplementary Tables). The sequences generated/analyzed in this study can be found in the GISAID database (https://gisaid.org/ (accessed on 11 February 2025)).

## Supplementary Materials

The following supporting information can be downloaded at: https://www.mdpi.com/article/10.3390/v17020270/s1, Table S1. Primers/probes used in this study. Table S2. GISAID virus/sequence identification/accession numbers of Bulgarian influenza strains analyzed in this study. Table S3. Amino acid substitutions identified in NA protein of influenza viruses A(H1N1)pdm09, A(H3N2), and B/Victoria lineage circulating in Bulgaria during the 2023–2024 season. Table S4. Amino acid substitutions identified in PB2, PB1, PA, NP, MP, and NS proteins of influenza viruses A(H1N1)pdm09, A(H3N2), and B/Victoria lineage circulating in Bulgaria during the 2023–2024 season.

## References

[1] Tabatabai J., Ihling C.M., Manuel B., Rehbein R.M., Schnee S.V., Hoos J., Pfeil J., Grulich-Henn J., Schnitzler P. (2023). Viral Etiology and Clinical Characteristics of Acute Respiratory Tract Infections in Hospitalized Children in Southern Germany (2014–2018). Open Forum Infect. Dis..

[2] Zhang L., Wang Y., Xu H., Hao L., Zhao B., Ye C., Zhu W. (2024). Prevalence of Respiratory Viruses in Children With Acute Respiratory Infections in Shanghai, China, From 2013 to 2022. Influenza Other Respir. Viruses.

[3] Babawale P.I., Guerrero-Plata A. (2024). Respiratory Viral Coinfections: Insights into Epidemiology, Immune Response, Pathology, and Clinical Outcomes. Pathogens.

[4] Tokars J.I., Olsen S.J., Reed C. (2018). Seasonal Incidence of Symptomatic Influenza in the United States. Clin. Infect. Dis..

[5] Uyeki T.M., Hui D.S., Zambon M., Wentworth D.E., Mon A.S. (2022). Influenza. Lancet.

[6] WHO The Burden of Influenza. https://www.who.int/news-room/feature-stories/detail/the-burden-of-influenza.

[7] CDC (2021). The Pink Book: Course Textbook—14th Edition. https://www.cdc.gov/pinkbook/hcp/table-of-contents/chapter-12-influenza.html?CDC_AAref_Val=https://www.cdc.gov/vaccines/pubs/pinkbook/flu.html.

[8] Caton A.J., Brownlee G.G., Yewdell J.W., Gerhard W. (1982). The antigenic structure of the influenza virus A/PR/8/34 hemagglutinin (H1 subtype). Cell.

[9] Wiley D.C., Wilson I.A., Skehel J.J. (1981). Structural identification of the antibody-binding sites of Hong Kong influenza haemagglutinin and their involvement in antigenic variation. Nature.

[10] Krystal M., Young J.F., Palese P., Wilson I.A., Skehel J.J., Wiley D.C. (1983). Sequential mutations in hemagglutinins of influenza B virus isolates: Definition of antigenic domains. Proc. Natl. Acad. Sci. USA.

[11] Skehel J.J., Stevens D.J., Daniels R.S., Douglas A.R., Knossow M., Wilson I.A., Wiley D.C. (1984). A carbohydrate side chain on hemagglutinins of Hong Kong influenza viruses inhibits recognition by a monoclonal antibody. Proc. Natl. Acad. Sci. USA.

[12] EU Case Definitions. https://ecdc.europa.eu/en/infectious-diseases-public-health/surveillance-and-disease-data/eu-case-definitions.

[13] Shu B., Kirby M.K., Davis W.G., Warnes C., Liddell J., Liu J., Wu K.H., Hassell N., Benitez A.J., Wilson M.M. (2021). Multiplex Real-Time Reverse Transcription PCR for Influenza A Virus, Influenza B Virus, and Severe Acute Respiratory Syndrome Coronavirus 2. Emerg. Infect. Dis..

[14] Shu B., Wu K.H., Emery S., Villanueva J., Johnson R., Guthrie E., Berman L., Warnes C., Barnes N., Klimov A. (2011). Design and performance of the CDC real-time reverse transcriptase PCR swine flu panel for detection of 2009 A (H1N1) pandemic influenza virus. J. Clin. Microbiol..

[15] Kodani M., Yang G., Conklin L.M., Travis T.C., Whitney C.G., Anderson L.J., Schrag S.J., Taylor T.H., Beall B.W., Breiman R.F. (2011). Application of TaqMan low-density arrays for simultaneous detection of multiple respiratory pathogens. J. Clin. Microbiol..

[16] Influenza Virus Characterization—Summary Europe, September 2024. https://www.ecdc.europa.eu/en/publications-data/influenza-virus-characterization-summary-europe-september-2024.

[17] Tamura K., Stecher G., Kumar S. (2021). MEGA11: Molecular Evolutionary Genetics Analysis Version 11. Mol. Biol. Evol..

[18] World Health Organization (2023). Recommended Composition of Influenza Virus Vaccines for Use in the 2023–2024 Northern Hemisphere Influenza Season.

[19] Blackburne B.P., Hay A.J., Goldstein R.A. (2008). Changing selective pressure during antigenic changes in human influenza H3. PLoS Pathog..

[20] Wilson J.R., Guo Z., Tzeng W.P., Garten R.J., Xiyan X., Blanchard E.G., Blanchfield K., Stevens J., Katz J.M., York I.A. (2015). Diverse antigenic site targeting of influenza hemagglutinin in the murine antibody recall response to A(H1N1)pdm09 virus. Virology.

[21] Sriwilaijaroen N., Suzuki Y. (2012). Molecular basis of the structure and function of H1 hemagglutinin of influenza virus. Proc. Jpn. Acad. Ser. B Phys. Biol. Sci..

[22] Govorkova E.A., Takashita E., Daniels R.S., Fujisaki S., Presser L.D., Patel M.C., Huang W., Lackenby A., Nguyen H.T., Pereyaslov D. (2022). Global update on the susceptibilities of human influenza viruses to neuraminidase inhibitors and the cap-dependent endonuclease inhibitor baloxavir, 2018–2020. Antiviral. Res..

[23] de Graaf M., Fouchier R.A. (2014). Role of receptor binding specificity in influenza A virus transmission and pathogenesis. EMBO J..

[24] Wang Q., Cheng F., Lu M., Tian X., Ma J. (2008). Crystal structure of unliganded influenza B virus hemagglutinin. J. Virol..

[25] Wang Q., Tian X., Chen X., Ma J. (2007). Structural basis for receptor specificity of influenza B virus hemagglutinin. Proc. Natl. Acad. Sci. USA.

[26] Rivas M.J., Alegretti M., Cóppola L., Ramas V., Chiparelli H., Goñi N. (2020). Epidemiology and Genetic Variability of Circulating Influenza B Viruses in Uruguay, 2012–2019. Microorganisms.

[27] Zhao P., Zhang Y., Wang J., Li Y., Wang Y., Gao Y., Zhao M., Zhao M., Tan H., Tie Y. (2024). Epidemiology of respiratory pathogens in patients with acute respiratory infections during the COVID-19 pandemic and after easing of COVID-19 restrictions. Microbiol. Spectr..

[28] Li H., Yang Y., Tao R., Shang S. (2024). Analyzing infections caused by 11 respiratory pathogens in children: Pre- and post-COVID-19 pandemic trends in China. J. Med. Virol..

[29] Zhao X., Gu Y., Tang X., Jiang C., Fang F., Chu W., Tao L., Zhang X., Chen M., Wu H. (2024). Whole-genome analysis of circulating influenza A virus (H3N2) strains in Shanghai, China from 2005 to 2023. Emerg. Microbes Infect..

[30] Lv G., Shi L., Liu Y., Sun X., Mu K. (2024). Epidemiological characteristics of common respiratory pathogens in children. Sci. Rep..

[31] Mostafa H.H., Fall A., Norton J.M., Sachithanandham J., Yunker M., Abdullah O., Hanlon A., Gluck L., Morris C.P., Pekosz A. (2024). Respiratory virus disease and outcomes at a large academic medical center in the United States: A retrospective observational study of the early 2023/2024 respiratory viral season. Microbiol. Spectr..

[32] Korsun N., Daniels R., Angelova S., Ermetal B., Grigorova I., Voleva S., Trifonova I., Kurchatova A., McCauley J. (2020). Genetic diversity of influenza A viruses circulating in Bulgaria during the 2018–2019 winter season. J. Med. Microbiol..

[33] Korsun N., Trifonova I., Dobrinov V., Madzharova I., Grigorova I., Christova I. (2023). Low prevalence of influenza viruses and predominance of A(H3N2) virus with respect to SARS-CoV-2 during the 2021–2022 season in Bulgaria. J. Med. Virol..

[34] Koutsakos M., Wheatley A.K., Laurie K., Kent S.J., Rockman S. (2021). Influenza lineage extinction during the COVID-19 pandemic?. Nat. Rev. Microbiol..

[35] Tramuto F., Maida C.M., Randazzo G., Previti A., Sferlazza G., Graziano G., Costantino C., Mazzucco W., Vitale F. (2024). Insights into Genetic and Antigenic Characteristics of Influenza A(H1N1)pdm09 Viruses Circulating in Sicily During the Surveillance Season 2023–2024: The Potential Effect on the Seasonal Vaccine Effectiveness. Viruses.

[36] Di Maio V.C., Scutari R., Forqué L., Colagrossi L., Coltella L., Ranno S., Linardos G., Gentile L., Galeno E., Vittucci A.C. (2024). Presence and Significance of Multiple Respiratory Viral Infections in Children Admitted to a Tertiary Pediatric Hospital in Italy. Viruses.

[37] Belazi S., Olsen S.J., Brown C., Green H.K., Mook P., Nguyen-Van-Tam J., Penttinen P., Lansbury L. (2021). Spotlight influenza: Laboratory-confirmed seasonal influenza in people with acute respiratory illness: A literature review and meta-analysis, WHO European Region, 2004 to 2017. Eurosurveillance.

[38] Siqueira B.A., Bredariol K.O., Boschiero M.N., Marson F.A.L. (2024). Viral co-detection of influenza virus and other respiratory viruses in hospitalized Brazilian patients during the first three years of the coronavirus disease (COVID)-19 pandemic: An epidemiological profile. Front. Microbiol..

[39] Mandelia Y., Procop G.W., Richter S.S., Worley S., Liu W., Esper F. (2021). Dynamics and predisposition of respiratory viral co-infections in children and adults. Clin. Microbiol. Infect..

[40] Shirreff G., Chaves S.S., Coudeville L., Mengual-Chuliá B., Mira-Iglesias A., Puig-Barberà J., Orrico-Sanchez A., Díez-Domingo J., Opatowski L., Valencia Hospital Surveillance Network for the Study of Influenza and Other Respiratory Viruses (VAHNSI) (2024). Seasonality and Co-Detection of Respiratory Viral Infections Among Hospitalised Patients Admitted With Acute Respiratory Illness-Valencia Region, Spain, 2010–2021. Influenza Other Respir. Viruses.

[41] Mauro M.V., Greco S., Pellegrini M., Campagna T., Caprino F., Elia N., Mastroianni A., Greco F. (2024). Epidemiology and Clinical impact of single and multi-viral respiratory infections in post-pandemic era. New Microbiol..

[42] Petat H., Corbet S., Leterrier B., Vabret A., Ar Gouilh M. (2024). Unravelling the acute respiratory infection landscape: Virus type, viral load, health status and coinfection do matter. Front. Cell Infect. Microbiol..

[43] Goka E.A., Vallely P.J., Mutton K.J., Klapper P.E. (2015). Single, dual and multiple respiratory virus infections and risk of hospitalization and mortality. Epidemiol. Infect..

[44] Lin G.L., Drysdale S.B., Snape M.D., O’Connor D., Brown A., MacIntyre-Cockett G., Mellado-Gomez E., de Cesare M., Ansari M.A., Bonsall D. (2024). Targeted metagenomics reveals association between severity and pathogen co-detection in infants with respiratory syncytial virus. Nat. Commun..

[45] Malveste Ito C.R., Moreira A.L.E., Silva P.A.N.D., Santos M.O., Santos A.P.D., Rézio G.S., Brito P.N., Rezende A.P.C., Fonseca J.G., Peixoto F.A.O. (2023). Viral Coinfection of Children Hospitalized with Severe Acute Respiratory Infections during COVID-19 Pandemic. Biomedicines.

[46] Scotta M.C., Chakr V.C., de Moura A., Becker R.G., de Souza A.P., Jones M.H., Pinto L.A., Sarria E.E., Pitrez P.M., Stein R.T. (2016). Respiratory viral coinfection and disease severity in children: A systematic review and meta-analysis. J. Clin. Virol..

[47] Asner S.A., Science M.E., Tran D., Smieja M., Merglen A., Mertz D. (2014). Clinical disease severity of respiratory viral co-infection versus single viral infection: A systematic review and meta-analysis. PLoS ONE.

[48] Piret J., Boivin G. (2022). Viral Interference between Respiratory Viruses. Emerg. Infect. Dis..

[49] Teluguakula N., Chow V.T.K., Pandareesh M.D., Dasegowda V., Kurrapotula V., Gopegowda S.M., Radic M. (2024). SARS-CoV-2 and Influenza Co-Infection: Fair Competition or Sinister Combination?. Viruses.

[50] Yunker M., Villafuerte D.A., Fall A., Norton J.M., Abdullah O., Rothman R.E., Fenstermacher K.Z.J., Morris C.P., Pekosz A., Klein E. (2024). Genomic evolution of influenza during the 2023–2024 season, the Johns Hopkins health system. J. Clin. Virol..

[51] Martínez J.L., Lemus N., Lai T.Y., Mishra M., González-Domínguez I., Puente-Massaguer E., Loganathan M., Francis B., Samanovic M.I., Krammer F. (2024). The immunodominance of antigenic site Sb on the H1 influenza virus hemagglutinin increases with high immunoglobulin titers of the cohorts and with young age, but not sex. Vaccine.

[52] Koel B.F., Mögling R., Chutinimitkul S., Fraaij P.L., Burke D.F., van der Vliet S., de Wit E., Bestebroer T.M., Rimmelzwaan G.F., Osterhaus A.D. (2015). Identification of amino acid substitutions supporting antigenic change of influenza A(H1N1)pdm09 viruses. J. Virol..

[53] Broberg E.K., Vukovikj M., Svartström O., Hasibra I., Riess M., Melidou A., Members of the ERLI-Net network (2024). Antigenic changes in influenza A(H3N2) driven by genetic evolution: Insights from virological surveillance, EU/EEA, week 40/2023 to week 9/2024. Eurosurveillance.

[54] Jin H., Zhou H., Liu H., Chan W., Adhikary L., Mahmood K., Lee M.S., Kemble G. (2005). Two residues in the hemagglutinin of A/Fujian/411/02-like influenza viruses are responsible for antigenic drift from A/Panama/2007/99. Virology.

[55] Eshaghi A., Duvvuri V.R., Li A., Patel S.N., Bastien N., Li Y., Low D.E., Gubbay J.B. (2014). Genetic characterization of seasonal influenza A (H3N2) viruses in Ontario during 2010–2011 influenza season: High prevalence of mutations at antigenic sites. Influenza Other Respir. Viruses.

[56] Chambers B.S., Parkhouse K., Ross T.M., Alby K., Hensley S.E. (2015). Identification of Hemagglutinin Residues Responsible for H3N2 Antigenic Drift during the 2014–2015 Influenza Season. Cell Rep..

[57] Popova L., Smith K., West A.H., Wilson P.C., James J.A., Thompson L.F., Air G.M. (2012). Immunodominance of antigenic site B over site A of hemagglutinin of recent H3N2 influenza viruses. PLoS ONE.

[58] Koel B.F., Burke D.F., Bestebroer T.M., van der Vliet S., Zondag G.C., Vervaet G., Skepner E., Lewis N.S., Spronken M.I., Russell C.A. (2013). Substitutions near the receptor binding site determine major antigenic change during influenza virus evolution. Science.

[59] Bedford T., Suchard M.A., Lemey P., Dudas G., Gregory V., Hay A.J., McCauley J.W., Russell C.A., Smith D.J., Rambaut A. (2014). Integrating influenza antigenic dynamics with molecular evolution. Elife.

[60] Perofsky A.C., Huddleston J., Hansen C.L., Barnes J.R., Rowe T., Xu X., Kondor R., Wentworth D.E., Lewis N., Whittaker L. (2024). Antigenic drift and subtype interference shape A(H3N2) epidemic dynamics in the United States. Elife.

[61] Lugovtsev V.Y., Vodeiko G.M., Strupczewski C.M., Ye Z., Levandowski R.A. (2007). Generation of the influenza B viruses with improved growth phenotype by substitution of specific amino acids of hemagglutinin. Virology.

[62] Chen R., Holmes E.C. (2008). The evolutionary dynamics of human influenza B virus. J. Mol. Evol..

[63] Vijaykrishna D., Holmes E.C., Joseph U., Fourment M., Su Y.C., Halpin R., Lee R.T., Deng Y.M., Gunalan V., Lin X. (2015). The contrasting phylodynamics of human influenza B viruses. Elife.

[64] Wang Y., Liu Y., Wang Y., Mai H., Chen Y., Zhang Y., Ji Y., Cong X., Gao Y. (2024). Clinical characteristics of outpatients with influenza-B-associated pneumonia and molecular evolution of influenza B virus in Beijing, China, during the 2021–2022 influenza season. Arch. Virol..

[65] Korsun N., Trifonova I., Madzharova I., Christova I. (2024). Resurgence of influenza with increased genetic diversity of circulating viruses during the 2022–2023 season. J. Med. Microbiol..

[66] Kim P., Jang Y.H., Kwon S.B., Lee C.M., Han G., Seong B.L. (2018). Glycosylation of Hemagglutinin and Neuraminidase of Influenza A Virus as Signature for Ecological Spillover and Adaptation among Influenza Reservoirs. Viruses.

