# Supplementary material for: Etiological Spectrum of Acute Respiratory Infections in Bulgaria During the 2023–2024 Season and Genetic Diversity of Circulating Influenza Viruses
Source: Viruses. 2025 Feb 16;17(2):270. doi: 10.3390/v17020270 (PMC11860199; doi:10.3390/v17020270)
Supplement: Supplementary file 1 [file viruses-17-00270-s001.zip › Supplementary-Table S1.pdf]

Table S1. Primers/probes used in this study

Assays	Target gene	Primer/probe sequence (5'–3')
Real time RT-PCR for		
Inf-A detection	M1	F: CAA GAC CAA TCY TGT CAC CTC TGA C<br>R: GCA TTY TGG ACA AAV CGT CTA CG<br>P: FAM-TGC AGT CCT (ZEN) CGC TCA CTG GGC ACG-(3IABkFQ)
Inf-B detection	NS2	F: TCC TCA AYT CAC TCT TCG AGC G<br>R: CGG TGC TCT TGA CCA AAT TGG<br>P: YakYel-CCA ATT CGA(ZEN) GCA GCT GAA ACT GCG GTG-(3IABkFQ)
SARS-CoV-2-detection	N	F: CTG CAG ATT TGG ATG ATT TCT CC<br>R: CCT TGT GTG GTC TGC ATG AGT TTA G<br>P: TexRd-XN/ATT GCA ACA/TAO/ ATC CAT GAG CAG TGC TGA CTC-(3IAbRQSp)
RSV detection	M	F: GGC AAA TAT GGA AAC ATA CGT GAA<br>R: TCT TTT TCT AGG ACA TTG TAY TGA ACA G<br>P: FAM-CTG TGT ATG TGG AGC CTT CGT GAA GCT-BHQ1
PIV-1 detection	HN	F: AGT TGT CAA TGT CTT AAT TCG TAT CAA T<br>R: TCG GCA CCT AAG TAA TTT TGA GTT<br>P: FAM-ATA GGC CAA AGA T(BHQ1)TG TTG TCG AGA CTA TTC CAA
PIV-2 detection	HN	F: GCA TTT CCA ATC TAC AGG ACT ATG A<br>R: ACC TCC TGG TAT AGC AGT GAC TGA AC<br>P: FAM-CCA TTT ACC T(BHQ1)AA GTG ATG GAA TCA ATC GCA AA
PIV-3 detection	HN	F: TGG YTC AAT CTC AAC AAC AAG ATT TAA G<br>R: TAC CCG AGA AAT ATT ATT TTG CC<br>P: FAM-CCC RTC TGT(BHQ1)TGG ACC AGG GAT ATA CTA CAA A
hMPV detection	F	F: CAA GTG TGA CAT TGC TGA YCT RAA<br>R: ACT GCC GCA CAA CAT TTA GRA A<br>P: FAM-TGG CYG TYA GCT TCA GTC AAT TCA ACA GA-BHQ1
RV detection	5’NCR	F: CP**A** GCC **T**GC GTG GC<br>R: GAA ACA CGG ACA CCC AAA GTA<br>P: FAM-TCC TCC GGC CCC TGA ATG YGG C- BHQ1
AdV detection	Hexon	F: GCC CCA GTG GTC TTA CAT GCA CAT C<br>R: GCC ACG GTG GGG TTT CTA AAC TT<br>P: FAM-TGC ACC AGA CCC GGG CTC AGG TAC TCC GA-BHQ1
BoV detection	NS1	F: TGC AGA CAA CGC YTA GTT GTT T<br>R: CTG TCC CGC CCA AGA TAC A<br>P: FAM-CCA GGA TTG GGT GGA ACC TGC AAA--BHQ1

Abbreviations: NCR, noncoding region; F, forward primer; R, reverse primer; P, probe; FAM, 6-carboxyfluorescein; BHQ, black hole quencher. Probe labeled at the 5'-end with the reporter molecule 6-carboxyfluorescein (FAM), with a ZEN quencher between the 9th and 10th nucleotide, and with 3′ Iowa Black FQ quencher (3IABkFQ) at the 3'-end. Underlining and boldface indicate a locked nucleic acid.
