# Supplementary material for: Etiological Spectrum of Acute Respiratory Infections in Bulgaria During the 2023–2024 Season and Genetic Diversity of Circulating Influenza Viruses
Source: Viruses. 2025 Feb 16;17(2):270. doi: 10.3390/v17020270 (PMC11860199; doi:10.3390/v17020270)
Supplement: Supplementary file 1 [file viruses-17-00270-s001.zip › Supplementary-Table S2.pdf]

Table S2. GISAID virus/sequence identification/accession numbers of Bulgarian influenza strains analyzed in this study

A(H1N1)pdm09 strains	Accession number	A(H3N2) strains	Accession number
A/Victoria/4897/2022	EPI_ISL_17830834	A/Darwin/9/2021	EPI_ISL_12109641
A/Bulgaria/149/2024	EPI_ISL_19193816	A/Bulgaria/2443/2024	EPI_ISL_19188242
A/Bulgaria/97/2024	EPI_ISL_19193815	A/Bulgaria/69/2024	EPI_ISL_19188241
A/Bulgaria/158/2024	EPI_ISL_19193814	A/Bulgaria/2402/2024	EPI_ISL_19188240
A/Bulgaria/279/2024	EPI_ISL_19193813	A/Bulgaria/514/2024	EPI_ISL_19188239
A/Bulgaria/2387/2024	EPI_ISL_19188236	A/Bulgaria/2398/2024	EPI_ISL_19188238
A/Bulgaria/2349/2024	EPI_ISL_19188234	A/Bulgaria/499/2024	EPI_ISL_19188237
A/Bulgaria/2348/2024	EPI_ISL_19188232	A/Bulgaria/493/2024	EPI_ISL_19188235
A/Bulgaria/2221/2024	EPI_ISL_19188227	A/Bulgaria/2507/2024	EPI_ISL_19188233
A/Bulgaria/2219/2024	EPI_ISL_19188226	A/Bulgaria/2502/2024	EPI_ISL_19188231
A/Bulgaria/2180/2024	EPI_ISL_19188225	A/Bulgaria/2261/2024	EPI_ISL_19188230
A/Bulgaria/2090/2024	EPI_ISL_19188223	A/Bulgaria/2256/2024	EPI_ISL_19188229
A/Bulgaria/1958/2024	EPI_ISL_19188221	A/Bulgaria/2222/2024	EPI_ISL_19188228
A/Bulgaria/1925/2024	EPI_ISL_19188220	A/Bulgaria/2091/2024	EPI_ISL_19188224
A/Bulgaria/1922/2024	EPI_ISL_19188218	A/Bulgaria/2020/2024	EPI_ISL_19188222
A/Bulgaria/1827/2024	EPI_ISL_19188216	A/Bulgaria/1884/2024	EPI_ISL_19188217
A/Bulgaria/1684/2024	EPI_ISL_19188213	A/Bulgaria/1719/2024	EPI_ISL_19188215
A/Bulgaria/1329/2024	EPI_ISL_19188209	A/Bulgaria/1718/2024	EPI_ISL_19188214
A/Bulgaria/1323/2024	EPI_ISL_19188207	A/Bulgaria/164/2024	EPI_ISL_19188212
A/Bulgaria/302/2024	EPI_ISL_19188115	A/Bulgaria/1331/2024	EPI_ISL_19188210
A/Bulgaria/238/2024	EPI_ISL_19188114	A/Bulgaria/1328/2024	EPI_ISL_19188208
A/Bulgaria/290/2024	EPI_ISL_19188113	A/Bulgaria/116/2024	EPI_ISL_19188206
A/Bulgaria/311/2024	EPI_ISL_19188112	A/Bulgaria/1075/2024	EPI_ISL_19188205
A/Bulgaria/312/2024	EPI_ISL_19188111	A/Bulgaria/1012/2024	EPI_ISL_19188204
A/Bulgaria/391/2024	EPI_ISL_19188110	A/Bulgaria/2696/2023	EPI_ISL_18962165
A/Bulgaria/439/2024	EPI_ISL_19188109	A/Bulgaria/2540/2023	EPI_ISL_18951906
A/Bulgaria/513/2024	EPI_ISL_19188108	A/Bulgaria/2703/2023	EPI_ISL_18951905
A/Bulgaria/574/2024	EPI_ISL_19188106	A/Bulgaria/1986/2024	EPI_ISL_19678127
A/Bulgaria/501/2024	EPI_ISL_19188105	A/Bulgaria/1874/2024	EPI_ISL_19678126
A/Bulgaria/561/2024	EPI_ISL_19188104	A/Bulgaria/2394/2024	EPI_ISL_19678115
A/Bulgaria/916/2024	EPI_ISL_19188103	A/Bulgaria/2310/2024	EPI_ISL_19678114
A/Bulgaria/571/2024	EPI_ISL_19188102	A/Bulgaria/2295/2024	EPI_ISL_19678113
A/Bulgaria/673/2024	EPI_ISL_19188098	A/Bulgaria/1819/2024	EPI_ISL_19678112
A/Bulgaria/674/2024	EPI_ISL_19188091	A/Bulgaria/1681/2024	EPI_ISL_19678111
A/Bulgaria/1015/2024	EPI_ISL_19188085	A/Bulgaria/1261/2024	EPI_ISL_19678110
A/Bulgaria/1016/2024	EPI_ISL_19188079	A/Bulgaria/628/2024	EPI_ISL_19678109
A/Bulgaria/1017/2024	EPI_ISL_19188078	A/Bulgaria/292/2024	EPI_ISL_19678108
A/Bulgaria/1327/2024	EPI_ISL_19188077		
A/Bulgaria/1211/2024	EPI_ISL_19188076		
A/Bulgaria/2705/2023	EPI_ISL_18962164		
A/Bulgaria/2257/2023	EPI_ISL_18951904		
A/Bulgaria/2347/2023	EPI_ISL_18951903		
A/Bulgaria/2346/2023	EPI_ISL_18951902		
A/Bulgaria/2368/2023	EPI_ISL_18951901		
A/Bulgaria/2453/2023	EPI_ISL_18951900		
A/Bulgaria/2630/2023	EPI_ISL_18951899		
A/Bulgaria/2494/2023	EPI_ISL_18951898		
A/Bulgaria/2553/2023	EPI_ISL_18951897		
A/Bulgaria/2660/2023	EPI_ISL_18951896		
A/Bulgaria/2682/2023	EPI_ISL_18951895		
A/Bulgaria/2591/2023	EPI_ISL_18951894		
A/Bulgaria/2592/2023	EPI_ISL_18951731		
A/Bulgaria/2594/2023	EPI_ISL_18951730		
A/Bulgaria/2625/2023	EPI_ISL_18951728		
A/Bulgaria/2663/2023	EPI_ISL_18951727		
A/Bulgaria/2695/2023	EPI_ISL_18951726		

A(H1N1)pdm09 strains	Accession number	B/Victoria lineage	Accession number
A/Bulgaria/2651/2023	EPI_ISL_18951725	B/Austria/1359417/2021	EPI_ISL_19313795
A/Bulgaria/2706/2023	EPI_ISL_18951724	B/Bulgaria/2406/2024	EPI_ISL_19188122
A/Bulgaria/756/2024	EPI_ISL_19680907	B/Bulgaria/2587/2024	EPI_ISL_18951723
A/Bulgaria/392/2024	EPI_ISL_19680904	B/Bulgaria/2579/2024	EPI_ISL_18951722
A/Bulgaria/333/2024	EPI_ISL_19680902	B/Bulgaria/1837/2024	EPI_ISL_19678125
A/Bulgaria/84/2024	EPI_ISL_19680899	B/Bulgaria/2413/2024	EPI_ISL_19678124
A/Bulgaria/2385/2024	EPI_ISL_19678107	B/Bulgaria/2406/2024	EPI_ISL_19678123
A/Bulgaria/2307/2024	EPI_ISL_19678106	B/Bulgaria/1985/2024	EPI_ISL_19678122
A/Bulgaria/1984/2024	EPI_ISL_19678105	B/Bulgaria/1981/2024	EPI_ISL_19678121
A/Bulgaria/1896/2024	EPI_ISL_19678104	B/Bulgaria/1966/2024	EPI_ISL_19678120
A/Bulgaria/1594/2024	EPI_ISL_19678103	B/Bulgaria/1854/2024	EPI_ISL_19678119
A/Bulgaria/1581/2024	EPI_ISL_19678102	B/Bulgaria/1850/2024	EPI_ISL_19678118
A/Bulgaria/1570/2024	EPI_ISL_19678101	B/Bulgaria/1845/2024	EPI_ISL_19678117
A/Bulgaria/1467/2024	EPI_ISL_19678100	B/Bulgaria/1814/2024	EPI_ISL_19678116
A/Bulgaria/1414/2024	EPI_ISL_19678099		
A/Bulgaria/1266/2024	EPI_ISL_19678098		
