# Supplementary material for: Etiological Spectrum of Acute Respiratory Infections in Bulgaria During the 2023–2024 Season and Genetic Diversity of Circulating Influenza Viruses
Source: Viruses. 2025 Feb 16;17(2):270. doi: 10.3390/v17020270 (PMC11860199; doi:10.3390/v17020270)
Supplement: Supplementary file 1 [file viruses-17-00270-s001.zip › Supplementary-Tables S3 and S4.pdf]

Table S3. Amino acid substitutions identified in NA protein of influenza viruses A(H1N1)pdm09 (n=74), A(H3N2) (n=37) and B/Victoria lineage (n=10) circulating in Bulgaria during the 2023-2024 season

Genetic groups	AA substitution	Number of strains (%)
A(H1N1)pdm09		
D.2	I40T	2 (2.7)
C.1.8, C.1.9, D.1 and D.2	D50N +CHO	72 (97.3)
D.2	N73S +CHO	2 (2.7)
C.1.8	V106N	5 (6.8)
C.1.8, C.1.9, D.1 and D.2	S200N	72 (97.3)
C.1.9	V234L	3 (4.1)
C.1.9	S247N	11 (14.9)
C.1.8 and C.1.9	I264T	45 (60.8)
C.1.9	K270R	10 (13.5)
C.1.9	M314I	12 (16.2)
C.1.8, C.1.9, D.1 and D.2	E382G	73 (98.6)
C.1.8	D392E	5 (6.8)
C.1.9	V394I	5 (6.8)
C.1.9	I396T	3 (4.1)
C.1.8	T438A	3 (4.1)
A(H3N2)		
All strains	S150H	37 (100)
	Y470H	37 (100)
J.1	I26T	4 (10.8)
J.1	S44P	3 (8.1)
J.2	E83G	12 (32.4)
J.2	K308R	7 (18.9)
J.1	S331G	4 (10.8)
J.1 and J.2	R400K	18 (48.6)
J.2	C446Y	31 (83.8)
B/Victoria lineage		
All strains	I459V	10 (100)
C.5.6	I45T	9 (90)
C.5.7	A47V	1 (10)
C.5.6	L73F	9 (90)
C.5.7	E338G	1 (10)
C.5.7	T372I	1 (10)
C.5.7	G378E	1 (10)
C.5.6	S397N	9 (90)

+CHO – gain of *N*-glycosylation site

Table S4. Amino acid substitutions identified in PB2, PB1, PA, NP, MP, and NS proteins of influenza viruses A(H1N1)pdm09, A(H3N2), and B/Victoria lineage circulating in Bulgaria during the 2023-2024 season

Gene segments	Viral proteins	Influenza A(H1N1)pdm09	Influenza A(H3N2)	Influenza B/Victoria
1	PB2	I81M, L475M	K62R, D107N, I147T, V410M	I396M, I465V, E467D
2	PB1	K57T, K757R	I179M, V517I	D51N, M65I
3	PA	V14I, M61I, L63I, R262K, K339N	G101E, N142K, V147I, I311M, Y321C, T402S, V407I, K605R	N158D, R357Q, I376M, S530T
5	NP	E14D, T22A, I33V, I136L, V217I, S450N, Q453P	M136L, D220E, R236K, I418L	A28T, N451S
7	M1	0	0	R105K, V354I
	M2	0	N24D, P25L, L54F, N82S	V105I
8	NS1	S212P	K26N, L33I, V60A, V82A, M124I, D152E, I171V, H207N	Y104H
	NEP	G67E, Q85H, I105L	R88K	0
